# Resonant Frequency Characteristics of a SAW Device Attached to Resonating Micropillars

**DOI:** 10.3390/s120403789

**Published:** 2012-03-23

**Authors:** N. Ramakrishnan, Harshal B. Nemade, Roy Paily Palathinkal

**Affiliations:** 1 School of Engineering, Monash University Sunway Campus, Jalan Lagoon Selatan, 46150 Bandar Sunway, Selangor Darul Ehsan, Malaysia; 2 Electronics and Electrical Engineering and Centre for Nanotechnology, Indian Institute of Technology Guwahati, Guwahati 781039, India; E-Mail: harshal@iitg.ernet.in; 3 Electronics and Electrical Engineering, Indian Institute of Technology Guwahati, Guwahati 781039, India; E-Mail: roypaily@iitg.ernet.in

**Keywords:** surface acoustic wave devices, resonance, microsensors

## Abstract

Recently we reported experimental and simulation results on an increase in resonance frequency of a SAW resonator caused by mass loading of micropillars made of SU-8, attached normal to the surface of the resonator. We concluded that SAW resonator and the SU-8 micropillars in unison form a system of coupled resonators. We have now extended this work and performed a finite element method simulation to study the resonance frequency characteristics of the SAW-based coupled resonator. In this paper we report the effect of the resonance frequency of the micropillars on the resonance frequency of the system of coupled resonators, and observe the coupling of micropillar resonance and the propagating SAW as described in the well known Dybwad system of coupled resonators.

## 1. Introduction

Surface acoustic wave (SAW) devices are widely used in sensor applications. The mass loading effect in SAW devices is one of the prime sensing principles in these types of sensors [1]. In our earlier work, we studied the mass loading effects of resonant structures such as high aspect ratio nanopillars attached to a SAW resonator and reported their application in designing highly sensitive SAW sensors [2,3]. More recently, we reported an increase in the resonance frequency of a SAW resonator caused by mass loading of SU-8 micropillars attached to the resonator [4]. We concluded that the SU-8 micropillars and the SAW resonator in unison form a coupled resonator system and for high values of contact stiffness the resonance frequency (*f*_0_) increases [4]. We have now extended our work on this SAW based coupled resonator to study the *f*_0_ of the system with pillars of different resonant frequency (*f*_r_) through finite element method (FEM) simulation. In this paper, we present the simulation results and discuss the coupled resonance between the SAW and the attached pillars with the help of Dybwad's explanation for systems of coupled resonators. Dybwad [5] was the first to report on coupled resonators consisting of gold particles of 10–50 μm diameter suspended on piezoelectric transducers and he observed an increase in resonance frequency of the resonator (*ω*_0_). Dybwad [5] concluded that the second resonator (gold particle) offers “inertial loading” to the quartz resonator when its resonance frequency is larger than the resonator's original resonance frequency *ω*_0_ and this results in a decrease in resonance frequency of the coupled resonator, while a weakly bonded particle offers “elastic loading” to the quartz resonator when the resonance frequency of the particles is smaller than *ω*_0_ and results in an increase in the resonance frequency of the coupled resonator [5,6]. The simulation methodology, observation on the resonance frequency shift caused by the SU-8 micropillar and discussions related to Dybwad's coupled resonance model are presented in the sections that follow.

## 2. Simulation Settings

**Figure 1.**
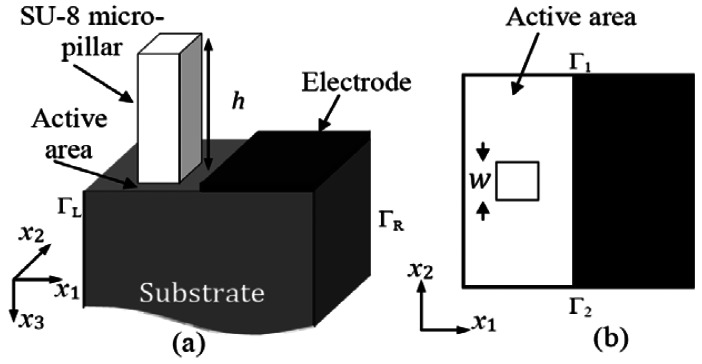
(**a**) A segment of SAW resonator geometry considered for the simulation, (**b**) top view of the segment (*x*_3_ = 0).

A one port SAW resonator consisting of a long interdigital transducer (IDT) with infinite number of fingers is considered for the simulation. The FEM model of piezoelectric material is explained in [7,8] and FEM simulations of SAW sensors are reported in [9–11]. The simulation of the SAW resonator is carried out using the piezoelectric module and SPOOLES solver of the commercial FEM software package COMSOL Multiphysics [8]. The substrate material and boundary conditions to model the SAW resonator are adapted from our earlier work [2–4]. By considering the periodic nature of the electrodes in an IDT, the SAW resonator is modeled as a segment of one pitch length (*p*) as shown in Figure 1 and the degrees of freedom (the displacement amplitude in all directions and voltage) of the right periodic boundary (Γ*_R_*) are set to be negative of those from the left periodic boundary (Γ*_L_*; see Figure 1). 3D simulations are performed with plane strain conditions and zero displacement constraint is provided along *x*_2_ direction to the boundaries Γ_1_ and Γ_2_. The bottom surface is fixed. The geometry of the segment (Figure 1) considered for the simulation is chosen to simulate a 39 MHz SAW resonator. Its dimensions are as follows: electrode width 21.5 μm, pitch (*p*) 43 μm, height of the substrate 430 μm, and thickness in *x*_2_ direction (aperture) 43 μm, leading to an active area of 21.5 μm × 21.5 μm. Triangular mesh is applied to the upper part of the substrate with minimum mesh size of 1 μm and rest of the SAW resonator is meshed with square mesh with dimension in the order of 4 μm. Initially eigen frequency analysis of the SAW resonator is performed and the resonance frequency without pillars (*f*_0_∣*_h_*_= 0_) of the SAW resonator is found to be 39.52702 MHz. Later a suitable resonant micropillar (in the shape of a square prism) made of SU-8 material of 8.6 μm × 8.6 μm cross-section is placed in the active area of the substrate (see Figure 1). Eigenfrequency analysis is performed for different values on height (*h*) of the micropillar varying from 0 to 50 μm and resonance frequency (*f*_0_) of the SAW resonator is recorded. SU-8 is a negative photoresist hard polymer. The SU-8 material properties such as Young's modulus of 4.02 GPa, density of 1,190 Kg/m^3^, and Poisson ratio of 0.22 are provided to the pillar in the simulation model. Further resonance frequency (*f_r_*) of the SU-8 micro pillar of different heights (*h*) is calculated from eigenfrequency analysis of the pillar alone. In the simulation model SU-8 has been chosen as a sample material for the micropillar to perform a general study on the resonance frequency characteristics of the SAW device attached with a resonant structure, however, it can be replaced with any suitable material of interest while designing a SAW based coupled resonator. It should be noted that we fabricated SU-8 micropillars on SAW resonators and studied their mass loading effect. We could observe coupled resonance phenomenon in the experiment and simulation results, and there was good agreement between them [2].

## 3. Results and Discussion

**Figure 2.**
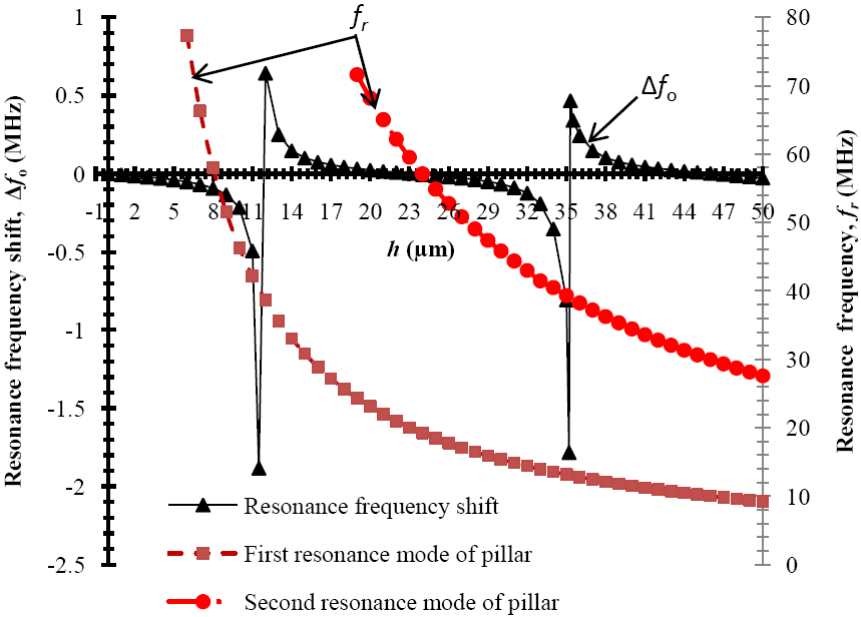
Plot of simulated results: Height of pillar versus resonance frequency shift, and resonance frequency of the pillar. The width of the pillar considered is 8.3 μm. Note that the resonance frequency of the SAW resonator is 39 MHz.

Figure 2 shows the plot of resonance frequency shift (Δ*f*_0_) of the SAW resonator for an increase in mass loading caused by SU-8 pillars of dimensions 8.6 μm × 8.6 μm × *h*. The Δ*f*_0_ is calculated by subtracting *f*_0_ from *f*_0_∣*_h_*_= 0_. It can be seen from the figure that as the pillar height is increased, the Δ*f*_0_ decreases and reaches a minimum value of −1.8 MHz at *h* = 11.5 MHz, however for *h* = 12 μm and *h* = 36 μm Δ*f_o_* increases, and reaches a value of 0.7 MHz and 0.5 MHz, respectively. As a whole, Δ*f_o_* is positive (increase in *f*_0_ from *f*_0_∣*_h_*_= 0_) for heights 12 μm < *h* < 23.4 μm, and 35 μm < *h <* 47 μm and Δ*f*_0_ is negative (decrease in *f*_0_ from *f*_0_∣*_h_*_= 0_) for heights 23. 4 μm < *h* < 35 μm, and for *h* > 47 μm. Figure 2 also includes the plot of resonance frequency (*f_r_*) of the pillar alone for different heights. The first two longitudinal vibration resonance modes of the micro pillar are shown. It can be seen that *f_r_* is between 10 MHz to 70 MHz for values of *h* ranging between 8 μm to 50 μm and 19 μm to 50 μm for the first and second resonance modes of the pillar respectively. Further comparing the *f_r_* and Δ*f*_0_ curves in Figure 2, when the resonance frequency of the pillar (*f_r_*) is closer to *f*_0_∣*_h_*_= 0_ the pillar offers negligible mass loading to the SAW device and resonance frequency shift tends to zero and reaches a positive value. This is a similar situation as reported in our earlier work [2,3]. The frequency characteristics of the SAW resonator upon mass loading by SU-8 micro pillar can be explained as follows by the coupling between the resonances of SU-8 micropillar and propagating SAW as pointed out by Dbywad [5]. The SU-8 micropillars resonate at their own resonance frequency and their vibrations couple to the acoustic mode of the SAW resonator. The SAW resonator and the SU-8 micro pillar together form a system of coupled resonators. It can be noted from Figure 2 that when the resonance frequency of the pillar *f_r_* is greater than *f*_0_∣*_h_*_= 0_ (that is *f_r_* > *f*_0_∣*_h_*_= 0_), Δ*f_o_* is negative and the pillars introduce inertial loading to the SAW resonator. When the resonance frequency of the pillar *f_r_* is less than *f*_0_∣ *_h_*_= 0_ (that is *f_r_* < *f*_0_∣*_h_*_= 0_), Δ*f_o_* is positive and the pillars introduce elastic loading to the SAW resonator. Further to visualize the resonance mode of the pillar and their effect on substrate surface, total displacement of the SAW resonator substrate with pillar and surface stress(*σ_x_*_3_) at the surface (*x*_3_ = 0, see Figure 1) of the substrate are recorded.

**Figure 3.**
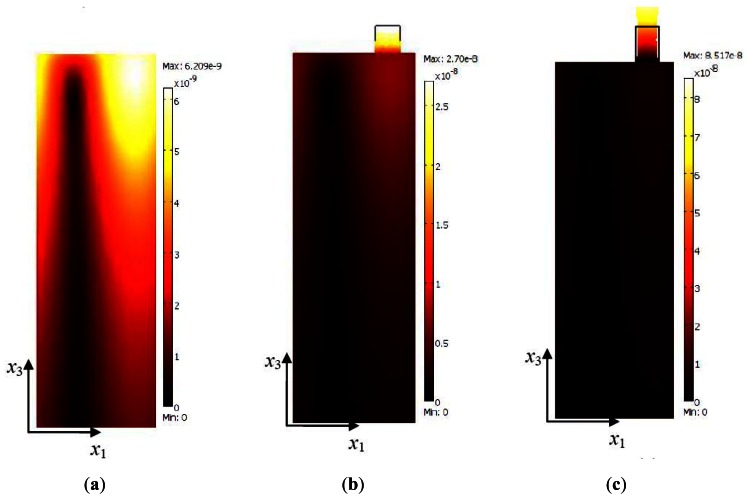

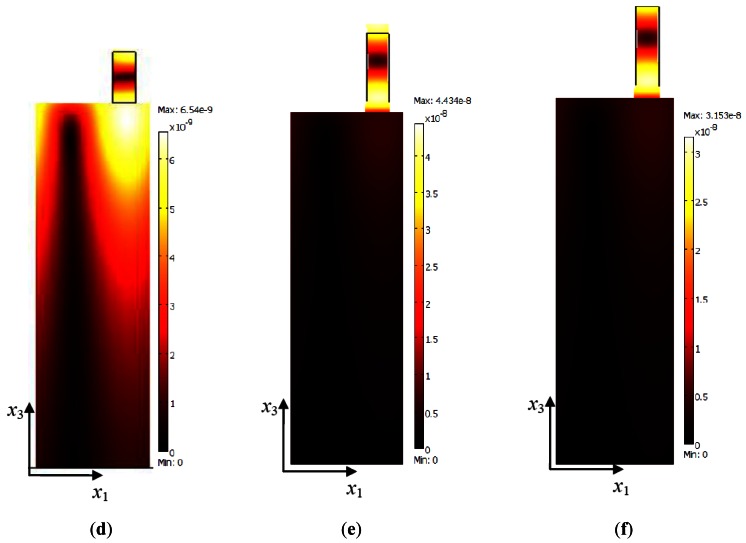
Simulation results: Total displacement in the substrate for the SAW resonator with SU-8 pillars of height (**a**) *h* = 0 μm (no pillar), (**b**) *h* = 10 μm, (**c**) *h* = 12 μm, (**d**) *h* = 23 μm, (**e**) *h* = 34 μm, and (**f**) *h* = 36 μm. The resonance mode of the pillar of different heights can be observed from the total displacement profile and deformation pattern of the pillars. For simplicity substrate depth up to 2λ along *x*_3_ is shown. In order to have a better visualization, the original deformation value is magnified 100 times and shown in the figure.

**Figure 4.**
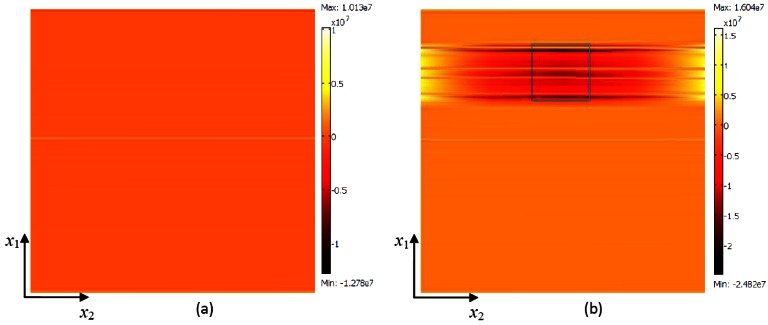

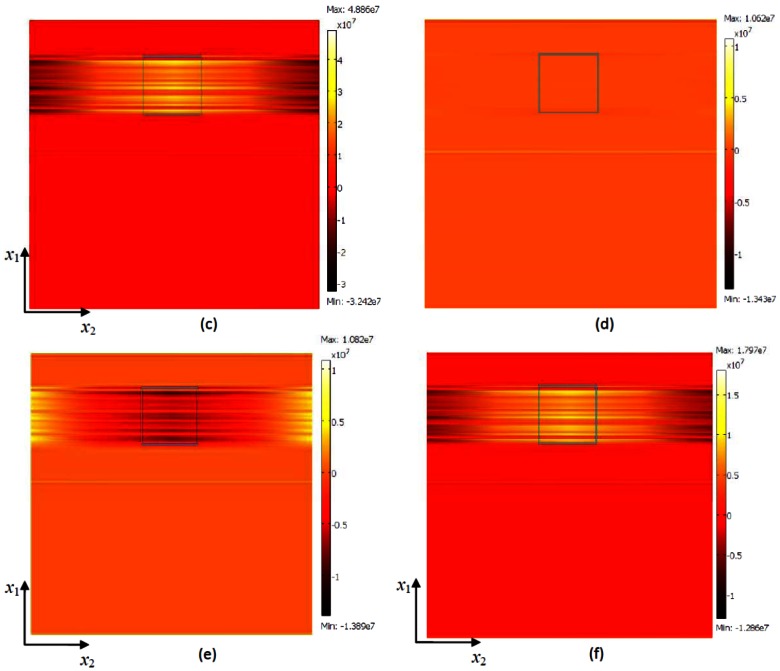
Simulation results: Normal stress *σ_x_*_3_ at the SAW resonator surface (at *x*_3_= 0 plane, the entire top surface of the segment considered in the simulation is shown) obtained for different heights of pillar (**a**) *h* = 0 μm (no pillar), (**b**) *h* = 10 μm, (**c**) *h* = 12 μm, (**d**) *h* = 23 μm, (**e**) *h* = 34 μm, and (**f**) *h* = 36 μm. The contact surface of the pillar is shown in square.

Total displacements of SAW is calculated by equation, $\text{total displacement}=\sqrt{{\left|u_{1}\right|}^{2}+{\left|u_{2}\right|}^{2}+{\left|u_{3}\right|}^{2}}$, where *u*_1_, *u*_2_, and *u*_3_ are the particle displacement in *x*_1_, *x*_2_, and *x*_3_ directions (see Figure 1), respectively. Total displacement of SAW substrate with pillars and corresponding *σ_x_*_3_ at surface *x*_3_ = 0 plane (see Figure 1) of a SAW resonator with pillars of typical heights *h* = 0 μm, 10 μm, 12 μm, 23 μm, 34 μm, and 36 μm are shown in Figure 3(a–f) and Figure 4 (a–f), respectively. Figure 3(b,c) shows the total displacement profiles of the pillars of resonance frequency in their first resonance mode and pillars offering inertial and elastic loading, respectively. Figure 3(d–f) shows the total displacement profile with typical heights of pillar of resonance frequency in their second resonance mode, where Figure 3(d,f) is a typical case of pillars offering elastic loading and Figure 3(e) is a typical case of pillars offering inertial loading to the SAW resonator. It can be seen from Figure 4, that the *σ_x_*_3_ value at the pillar contact surface (the pillar footprint is indicated by a square geometry) is negative when the pillar offers inertial loading and positive when the pillar offers elastic loading. It should be noted that darker color indicates minimum stress value and lighter color indicates maximum stress value. For a typical case of inertial loading height of *h* = 10 μm, as seen from Figure 4(b) the surface stress profile is darker (reddish to reddish brown) within the pillar contact area and *σ_x_*_3_ has a minimum value of −2.48 MPa, indicating a compressive stress at the pillar contact surface. On a contrary, for a typical case of elastic loading (*h* = 36 μm, Figure 4(f)) the stress profile is lighter (yellowish-whitish) within the square box area and *σ_x_*_3_ value is at maximum of 1.79 MPa, indicating tensile stress at the pillar contact surface. It should be noted that the displacement profile of SAW resonator with SU-8 pillar of height *h* = 23 μm (Figure 3(d)) is more or less similar to the displacement profile obtained for a SAW resonator without a pillar (Figure 3(a)), further the *σ*_s_ profile for the case of SAW resonator without the pillar and SAW resonator with a pillar of *h* = 23 μm is almost similar and the value of *σ*_s_ is approximately zero. It can be seen also be seen from Figure 2 that Δ*f*_0_ ∣*_h_*_= 23μm_ is 16 Hz, which is a negligible resonance frequency shift. Thus one can conclude that at *h* = 23 μm the SU-8 micropillars offer almost zero mass loading to the SAW resonator.

**Figure 5.**
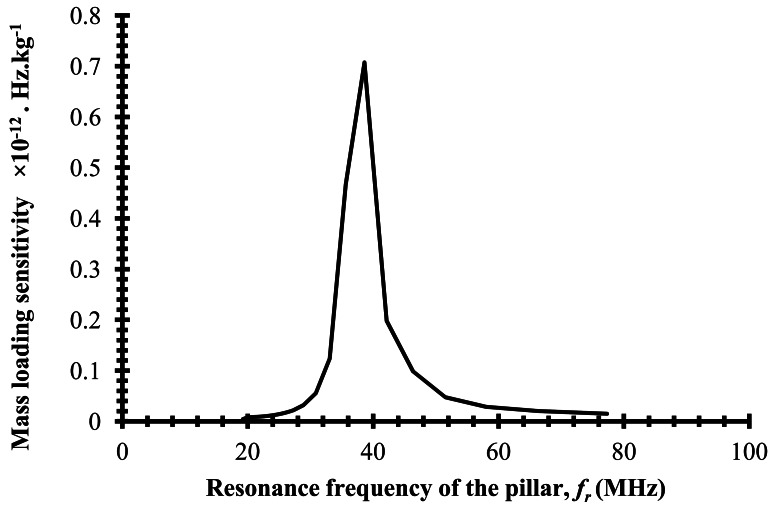
Plot of mass loading sensitivity versus resonance frequency of the pillar.

The present work will be of interest to sensor community readers in designing highly sensitve SAW sensors using resonant structures as sensing medium. In our earlier study we observed that the sensitivity with the resonant pillars is at least 10 times that obtained by using a thin film as the sensing medium [2,3]. Figure 5 shows the plot of mass loading sensitivity versus resonance frequency of the micropillar. Mass loading sensitivity is calculated as change in Δ*f*_0_ per μm increase in *h*. It can be seen from Figure 5 that the maximum sensitivity is obtained when *f_r_* = 38.6 MHz, which is closer to *f*_0_∣*_h_*_=0_. Thus highly sensitive SAW sensors based on coupled resonance can be designed by choosing a sensing medium made of a resonant structure that has a resonance frequency close to the resonance frequency of the SAW resonator.

## 4. Conclusions

FEM simulation of a system of coupled resonators made of a SAW resonator and aspect ratio micropillars is performed and the resonance frequency characteristics of the coupled resonator are studied for different values of pillar resonance frequencies. The micropillars offer inertial loading to the SAW resonator when *f_r_* > *f*_0_∣*_h_*_= 0_ and elastic loading to the SAW resonator when *f_r_* < *f*_0_∣*_h_*_= 0_. The resonance frequency characteristics observed in the simulation were in agreement with Dybwad's coupled resonance model. The SU-8 pillars used in the study can be replaced with a suitable sensing medium of resonant structures and can be used in sensing applications.
